# Off-label use of intravenous rifampicin during surgery: analysis of Australian surveillance data and retrospective audit at a tertiary hospital

**DOI:** 10.1017/ash.2025.71

**Published:** 2025-04-23

**Authors:** Nadine T. Hillock, Edward Raby, Matthew Rawlins

**Affiliations:** 1 School of Public Health, University of Adelaide, Adelaide, SA, Australia; 2 Department of Infectious Diseases, Fiona Stanley Hospital, Murdoch, WA, Australia; 3 PathWest Laboratory Medicine, Perth, WA, Australia; 4 Department of Pharmacy, Fiona Stanley Hospital, Murdoch, WA, Australia

## Abstract

**Objective:**

To investigate the use of rifampicin vials in Australian operating theaters (OT) to determine the method of administration and rationale for use.

**Methods:**

Retrospective (2022 and 2023) OT usage data for rifampicin 600 mg vials were analyzed to compare trends in use between Australian hospitals and between jurisdictions. An audit of rifampicin vials used in OT during 2023 was conducted at a large tertiary hospital.

**Results:**

Fifty-nine of 248 hospitals (24%) contributing data to the National Antimicrobial Utilisation Surveillance Program recorded OT use of rifampicin vials during 2022 and 2023. Excluding hospitals with no usage, the median use was 7 vials/annum/per hospital (IQR: 2–32). A wide variation in use was seen between Australian states and territories. An audit of OT use in 2023 at a large tertiary hospital found poor documentation of topical use; in most cases, documentation was in the operation note only, with no documentation on the medication charts, medical notes, or the anesthetic record. Of 33 rifampicin vials used in 2023, documented topical use was identified for 10 individual patients only, 4 of whom had a confirmed *Staphylococcus aureus* infection (1 methicillin-resistant and 3 methicillin-susceptible).

**Conclusion:**

Off-label, topical use of rifampicin during surgery is not uncommon in some Australian hospitals despite limited evidence of safety or efficacy. Given the potential for resistance, surgical use of rifampicin should be restricted to a named-patient basis, under the guidance of an infectious disease specialist/clinical microbiologist. Documentation of all medication use is recommended for patient safety.

## Introduction

The incidence of antimicrobial-resistant infections is increasing globally, with a recent systematic analysis forecasting that the antimicrobial resistance (AMR) burden will increase to 1.9 million attributable deaths and 8.2 million associated deaths by 2050.^1^ Methicillin-resistant *Staphylococcus aureus* (MRSA) was the organism attributed to the most AMR-related deaths in 2019, estimated at more than 100,000.^2^

Overuse and inappropriate use of antimicrobials are the key drivers of AMR. Results of the 2022 Surgical National Antimicrobial Prescribing Survey (SNAPS), a point prevalence survey assessing appropriateness of antimicrobial use in Australia, suggests that the overall rate of appropriate antimicrobial prophylaxis per surgical episode remains low (55.3%), with appropriateness of prescribing for some surgical craft groups being as low as (34%).^3^ Prolonged duration of topical antimicrobials used post-procedurally was a common reason for inappropriate use.^3^ With the exception of some ophthalmic surgical procedures, routine use of topical antimicrobials on surgical sites is not recommended as it contributes to the emergence of AMR.^4^

National guidelines do not recommend the use of antimicrobials as irrigations, pastes, or washes for surgical prophylaxis as there is limited high-quality evidence of superior outcomes compared with standard-of-care systemic surgical prophylaxis.^4,5^ Additionally, the practice of soaking vascular grafts or other implants or prostheses is not recommended as surgical prophylaxis, with most published studies reporting the outcomes of antibiotic-soaked grafts being case reports or case series.^6,7^ Some systematic reviews report improved patient outcomes with antibiotic soaking of endovascular stent grafts; however, the articles included in the reviews are predominantly low-quality (level IV) studies with a high risk of bias.^8^ Furthermore, none of these published studies include an analysis of any emergent AMR post-surgery.

Rifampicin has in vitro activity against gram-positive organisms embedded in biofilm and so may have a specific role in the treatment and prevention of implant-associated infections.^9^ Commercially available rifampicin-embedded devices have been marketed, for example, bioabsorbable envelopes for pacemakers.^10^ Other uses include rifampicin-impregnated catheters^11^ and endovascular stent grafts;^12^ however, some studies have shown that flushing or soaking of endografts with rifampicin is associated with an increased risk of emergent resistance.^13^ A number of flaws were identified in the trial evidence that supported the marketing authorization of rifampicin- and minocycline-embedded envelopes for pacemakers; more cases of bacteremia or endocarditis were reported in the envelope group compared to the placebo group, and no antibiotic susceptibilities were reported for any of the reported infections in either the intervention or placebo arms.^14^ Despite rifampicin’s potential to increase the risk of resistance, none of the follow-up studies have investigated AMR in patients with the implanted envelopes.

Devices or implants that are embedded with antibiotics usually fall outside the scope of the antimicrobial stewardship (AMS) teams because they are classified as surgical devices by regulatory bodies. Clinical pharmacists may be unaware that a patient has an implanted device containing and/or eluting rifampicin. Systemic absorption of antibiotics embedded in devices depends on a number of factors, including the lipophilicity of the antibiotic, the device size, the local dose or concentration, and the vascularity of the device location. The manufacturer of rifampicin-embedded envelopes suggests that while local rifampicin concentrations are high, systemic absorption is minimal;^15^ however, the kinetic studies to support this have not been published nor independently validated. There have been many case reports of rifampicin induction of hepatic enzymes affecting warfarin titration post-surgery or other medicines that are metabolized by hepatic cytochrome P450 (CYP) enzymes;^16–18^ however, there is a lack of data on the impact of locally administered rifampicin on hepatically metabolized medicines with a narrow therapeutic index.

Direct administration of antibiotic powders onto surgical wounds has been reported in the literature,^19,20^ and the practice has been anecdotally reported at some hospitals in Australia, particularly using vancomycin powder. Intravenous vancomycin is recommended as additional cover for surgical prophylaxis in patients at high risk of MRSA;^5^ however, there is a lack of strong evidence to support the topical use of vancomycin or other antibiotic powders directly onto surgical wounds. As well as published reports of direct application of antibiotic powders to surgical wounds, restricted antimicrobials have also been added to bone cements^21^ or used intra-articularly as prophylaxis^22^ or to “decontaminate” infected implants.^23^ Systemically administered rifampicin can be an effective adjunct in the treatment of prosthetic joint infections;^24^ however, the addition of rifampicin to bone cement is not recommended as it has been demonstrated to interfere with the curing of polymethyl methacrylate cements, significantly reducing the joint mechanical strength.^25,26^ Furthermore, the elution of rifampicin from bone cement is poor, resulting in subtherapeutic delivery of antibiotic into the infected joint.^27^

While there are frequent anecdotal reports of off-label use of last-line antimicrobials during surgery, for example, as irrigations, washes, or graft-soaking, the extent and scope of this practice in Australia are currently unclear. National-level surveillance data measuring antimicrobial use in the theater and recovery setting are volume-based^28^ and deidentified; therefore, patient-level, detailed information on the extent of off-label use of restricted antimicrobials is lacking. Inconsistent documentation of off-label use of antimicrobials in bone cement has been previously highlighted, with usage documented predominantly in the prosthetic record (39%) or the operation report (21%) but rarely on the drug chart (6%).^21^

Rifampicin accounted for 0.2% of the total volume of antibacterials used in Australian operating theaters (OT) (based on pharmacy dispensing and distribution data reported to the National Antimicrobial Utilisation Surveillance Program (NAUSP) 2021.^29^ Although this is not a large proportion of all antibacterials used in theater, by volume, this use is noteworthy given that rifampicin is not recommended for surgical prophylaxis.

The aim of this study was to investigate the comparative use of rifampicin vials in the theater setting between hospitals contributing to the national surveillance program and to conduct an audit of use at 1 large tertiary hospital to understand the surgical indications for use at this hospital in 2023.

## Method

Annual usage data for 2022 and 2023 of rifampicin 600 mg vials, distributed from the pharmacy department to the OT, were retrieved from the NAUSP on March 19, 2024. Usage was compared between jurisdictions and hospitals to determine trends in use.

A retrospective audit of rifampicin vial use in theater during 2023 was conducted at a large tertiary hospital. Transaction data for rifampicin vials were extracted from the automated dispensing cabinet (ADC) located in theater to identify the patients for whom rifampicin vials were used. Where no patient details were included in the ADC data, theater lists corresponding to the dates that rifampicin vials were dispensed from the ADC were retrieved to identify additional patients for whom rifampicin may have been administered, and corresponding medical records were searched. Data fields collected included the type of surgical procedure, the location of documentation in the medical notes, the method of administration, and the amount of rifampicin used. Additionally, the MRSA colonization status of the patient and whether rifampicin was used in the setting of prophylaxis or active infection, along with the causative organism, were recorded.

### Ethics statement

National surveillance data retrieved for the purpose of this study were non-identifiable and involved negligible risk, therefore meeting the conditions for exemption from ethical review.

The audit was approved as a quality improvement activity by the institutional Quality Improvement Committee (GEKO #54969) and considered exempt from requiring review by the Human Research Ethics Committee.

## Results

### Comparative use of rifampicin vials in theater and recovery between hospitals

**Figure 1.**
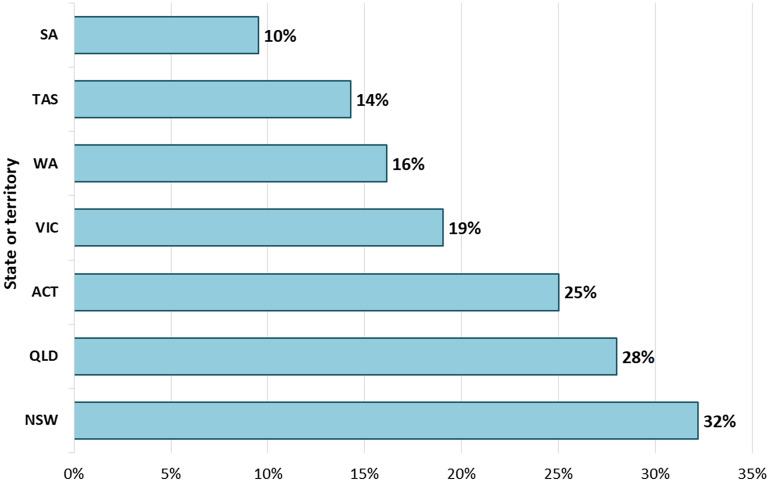
Proportion of hospitals, by state/territory, contributing data to NAUSP that reported use of rifampicin 600 mg vials in theatre, 2022-2023. Note: No hospitals in the Northern Territory reported use of rifampicin in theatre in 2022-2023. ACT, Australian Capital Teritory; NAUSP, National Antimicrobial Utilisation Surveillance Program; NSW, New South Wales; QLD, Queensland; SA, South Australia; TAS, Tasmania; VIC, Victoria; WA, Western Australia.

A total of 248 hospitals contributed antimicrobial usage data for theater and recovery in the 2-year period from 2022 to 2023. Fifty-nine of the 248 Australian hospitals (24%) reported the use of rifampicin vials in theater. Figure 1 shows the percentage of hospitals in each state or territory that reported the use of rifampicin vials in theater. There was no reported theater use of rifampicin vials in Northern Territory hospitals enrolled in the NAUSP, whereas 28 of the 87 New South Wales hospitals (32%) reported use of rifampicin vials in theater.

**Figure 2.**
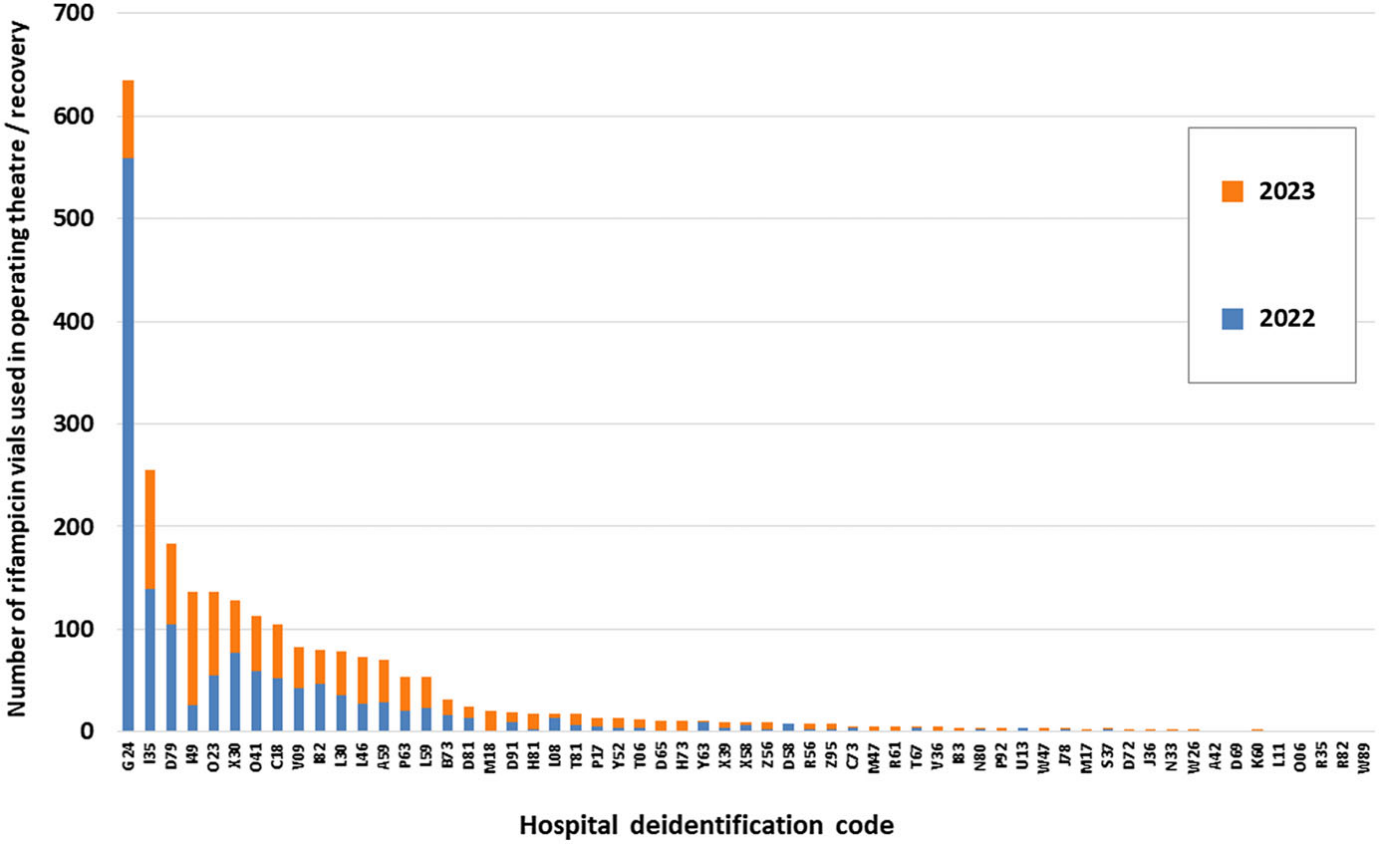
Annual theatre use of rifampicin 600 mg vials by Australian hospital, 2022-2023. Note: Hospitals with no reported theatre use of rifampicin vials not included. Each de-identified code represents a hospital or healthcare facility.

Figure 2 illustrates the wide variation in surgical rifampicin use between hospitals contributing data to the NAUSP in 2022 and 2023. In general, the usage rate was not high—the median use per hospital was 7 vials per annum (excluding those hospitals that reported no use). Eight of the 59 hospitals used more than 100 vials over the 2-year period, with 1 outlier hospital reporting the use of 635 vials.

### Results of audit of the use of rifampicin vials at a single principal referral hospital

**Figure 3.**
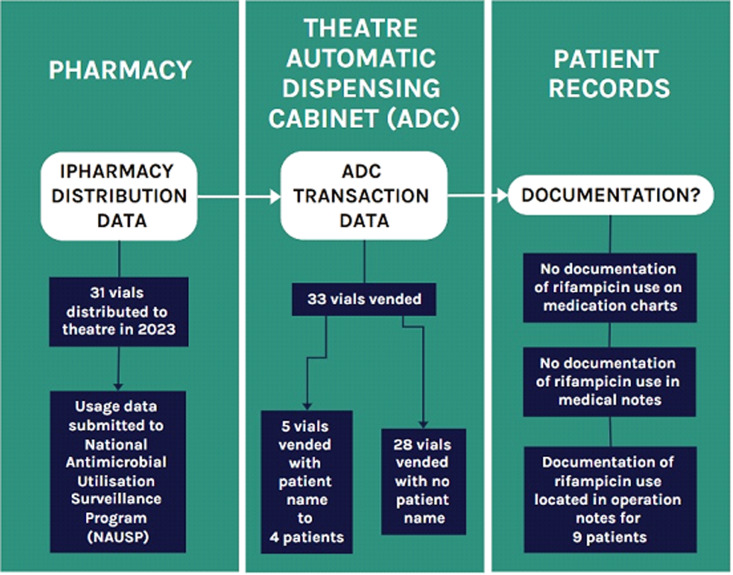
Flow diagram of audit of rifampicin vials used in theater (2023).

Figure 3 below illustrates the audit process undertaken for rifampicin use in theater in 2023 at a large Principal Referral hospital.

Of the 33 rifampicin vials vended from the ADC during 2023, 5 vials were dispensed to 4 patients, and 28 (84.8%) vials were dispensed with no patient name. In total, 10 patients were identified who had documented use of rifampicin during their surgery. For 9 of the 10 patients, rifampicin use was documented in the operation note, and the tenth patient was identified from the ADC record only, with no documentation included in the patient notes. In all 10 cases, there was no documentation of rifampicin use on the medication charts, the medical notes, or in the anesthetic record.

In total, documentation was identified for the use of 11 vials (33.3%), based on the assumption that at least 1 vial was used for each of the 10 patients, with 1 patient known to have received at least 2 vials (from the ADC report). The majority of recorded use was for treatment of infection; however, for 2 patients undergoing vascular procedures, the topical use of rifampicin was for prophylaxis. Only 1 of the 10 patients was colonized with MRSA.

Table 1 provides the details of the method of documentation, the type of procedures, the MRSA status of the patient, the method of administration, and whether the use was for prophylaxis or treatment of a known infection with an identified pathogen.

**Table 1.** Summary of patients with documented off-label administration of rifampicin vials

Patient no.	Age/sex	Documentation			Amount of rifampicin dispensed from ADC	Amount (mg) of rifampicin administered	Known or suspected infection or prophylaxis	Direct topical application of rifampicin powder	ID/micro advice sought for rifampicin use	Description (text) of documented use of rifampicin	Operation	MRSA colonized	Systemic antimicrobial prophylaxis	Other off-label antibiotic use documented
		In medical notes	On drug chart	In anesthetic record/operation notes/prosthetic record										
1	71M	No	No	Operation note	Not known	Not documented	Prophylaxis	Yes	No	“Rifampicin to both groins”	Right axillo-bifemoral graft and bilateral SFA thrombectomy	No	cefazolin 2 g × 2	. .
2	77F	No	No	Operation note	Not known	Not documented	Prophylaxis	Yes	No	“Rifampicin powder to both territories”	Left femoral endarterectomy and fem-above-knee bypass with prosthetic	No	cefazolin 2 g then 1 g	. .
3	55M	No	No	Operation note	1 × 600 mg vial	Not documented	Infection (MRSA)	Wash	No	“Thorough lavage with saline and wash with rifampicin”	Emergency AV replacement (Magna Ease bioprosthesis) for MRSA PVIE	Yes	vancomycin 2 g then vancomycin 1 g/rifampicin 450 mg/ciprofloxacin 500 mg q12h post-op	. .
4	29M	No	No	No	2 × 600 mg vials	Not documented	Infection *(Cutibacterium acnes)*	Unknown	No	Nil	Redo Bentall procedure with hemiarch replacement	No	vancomycin 1.5 g/cefazolin 2 g × 2, then vancomycin/piptaz per op then daptomycin post-op	. .
5	72F	No	No	Operation note	1 × 600 mg vial	Not documented	Infection (MSSA)	Wash	No	“Rifampicin wash”	Emergency mitral valve replacement for MSSA PVIE	No	cefazolin 2 g × 2 then flucloxacillin, ciprofloxacin, rifampicin	. .
6a	74M	No	No	Operation note	1 × 600 mg vial	Not documented	Infection *(Streptococcus mitis)*	Wash	No	“Rifampicin wash”	Aortic root replacement (modified Bentall with bioprosthesis), tricuspid valve annuloplasty for PVIE	No	cefazolin/vancomycin	. .
6b	74M	No	No	Operation note	. .	. .	. .	. .	. .	. .	Re-open for clot	No	cefazolin	. .
7a	60M	No	No	Operation note	Not known	Not documented	Infection *(Streptococcus agalactiae)*	Yes	No	“Topical hydrogen peroxide and rifampicin (to mitral valve)”	Mitral valve repair for *S. agalactiae* MVIE – vegetation resection and annual base of infection debridement	No	amoxicillin, cefazolin 2 g × 2	Vancomycin powder – direct application
7b	60M	No	No	Operation note	. .	. .	. .	. .	. .	. .	Emergency re-opening and evacuation of mediastinal hematoma	No	cefazolin 2 g, vancomycin 1.5 g	. .
8	71F	No	No	Operation note	Not known	Not documented	Infection *(E. faecalis)*	Yes	No	“Sutures placed through annulus, brought through valve cuff, secured down with Coreknot, topical rifampicin”	Mitral valve replacement for *E. faecalis* IE	No	cefazolin 2 g × 2, amoxicillin/gentamicin	Vancomycin powder to the sternum
9	65M	No	No	Operation note	Not known	Not documented	Infection (MSSA)	Yes	No	“St Jude Epic bioprosthesis rinsed with rifampicin”	Mitral valve replacement	No	cefazolin 2 g × 2, flucloxacillin 2 g × 2	. .
10	44F	No	No	Operation note	Not known	Not documented	Infection (MSSA)	Yes	No	“Prosthetic valve excised, and annulus extensively debrided and washed, and rifampicin and peroxide applied. All foreign material removed”	Tricuspid prosthetic valve replacement for PVIE	No	Vancomycin 1.5 g, cefazolin 2 g × 2	. .

Note. SFA, superficial femoral artery; MRSA, methicillin-resistant *Staphylococcus aureus*; PVIE, pulmonary valve infective endocarditis; MSSA, methicillin-susceptible *Staphylococcus aureus*; *MVIE*, *mitral valve infective endocarditis*.

## Discussion

Approximately one quarter of hospitals reporting to the NAUSP reported surgical use of rifampicin; however, in most cases, the number of vials used in the OT per annum was low (median use 7 vials/annually). A small number of outlier hospitals (n = 8) reported using more than 100 rifampicin vials in theater over the 2-year period from January 2022 to December 2023.

The audit of theater use of rifampicin vials at a large teaching hospital illustrated a lack of routine documentation when vials are used topically as a powder or a wash, with no documentation found on medication charts, the anesthetic record, or in the medical notes. The scope of this practice may be more substantial than noted here; despite the reported use of 2,526 vials of rifampicin in OT in hospitals reporting antimicrobial usage data to the NAUSP in 2023, there were very few instances of rifampicin use reported in the SNAPS, the annual point prevalence survey of surgical antimicrobial use in Australian hospitals.^30^ This retrospective audit at a large teaching hospital illustrates that if documentation is lacking, the use cannot be captured in national audits. Two of the 10 patients who received documented use of topical rifampicin were also administered vancomycin powder topically. While this study focused on rifampicin, it is possible that other antimicrobials are used off-label during surgery, given that documentation of that use is inconsistent and not in medication charts.

In general, rifampicin has poor permeability across the skin when applied topically due to its relatively large molecular size; however, when applied directly into a surgical wound, the skin barrier is bypassed, potentially allowing for greater systemic absorption. Rifampicin is a potent inducer of several CYP enzymes, including CYP3A4, CYP2C9, CYP2C19, and CYP1A2.^31^ Induction of these enzymes can lead to clinically significant reductions in therapeutic concentrations of drugs metabolized by those enzymes, potentially seriously affecting patient outcomes.^16,31^ Rifampicin use into a wound may therefore have unexpected clinical outcomes due to systemic absorption of rifampicin interacting with other medications, and the lack of documentation of rifampicin use does not allow for consideration of dose adjustments of interacting drugs.

The evidence to guide antimicrobial treatment of staphylococcal infection of prosthetic valves is poor, and associated mortality rates are high. High-dose intravenous flucloxacillin is recommended where methicillin-susceptible *Staphylococcus aureus* (MSSA) is the causative organism, with vancomycin recommended for MRSA.^5^ Some international guidelines^32^ suggest adding rifampicin (and gentamicin); however, the data to support this are poor, and the risk of adverse events is increased.^5^ Three of the 10 patients had MSSA prosthetic valve infective endocarditis, and 1 was infected with MRSA, with the remaining 6 patients negative for MRSA or MSSA colonization. Rifampicin use, particularly when used as monotherapy and early in the infection course when there is a higher organism burden, is associated with the rapid emergence of resistance; there is also potential for antagonistic activity of rifampicin combinations against replicating bacteria. For this reason, European endocarditis guidelines^33^ recommend waiting 3 days before introducing adjunctive rifampicin. The most recent Australian surveillance data report for MRSA susceptibility reported that all MRSA isolates tested were susceptible to vancomycin, but 1.5% were resistant to rifampicin.^34^

Similar to the topical administration of rifampicin directly into the surgical field, there is also no high-quality evidence to support the washing or soaking of grafts or prostheses with rifampicin. A 2019 in vitro study showed that rifampicin-soaked grafts demonstrated inferior 7-day bactericidal efficacy compared to a vascular graft containing silver and triclosan.^35^ Additionally, the study confirmed that rifampicin soaking exposed patients to an increased risk of hosting rifampicin-resistant bacteria.^35^

Off-label, topical use of antibiotic powders has been reported in the literature for other antimicrobials, including vancomycin powder. Most of the published data are case reports only, and there are limited data on patient outcomes, with no data available on the impact on AMR. Concerns have been raised regarding the safety of directly applying acidic antibiotics such as vancomycin into the surgical field and the possible consequences on wound healing. Case reports have been published, suggesting a risk of direct tissue damage and possible impairment of healing after the direct application of vancomycin powder to a wound due to the acidity of the powder.^36–38^ Vancomycin irrigations are increasingly used to reduce the risk of deep infections after breast reconstruction with implants; however, a recent retrospective review of 1,508 patients found no significant difference in infection rates with vancomycin irrigations.^39^

This audit of the use of rifampicin vials in the OT at a large principal referral hospital illustrated the variable methods of off-label use and the limited documentation associated with that use. It highlights an area where AMS teams can focus efforts to improve the governance over restricted antibiotics contained in ADCs, as well as the oversight of use and documentation of that use.

The audit of rifampicin use was limited by the lack of documentation. It is therefore possible that rifampicin use was documented for other patients, but capture of that data was difficult due to a lack of patient details included in the ADC transactions report. All efforts were made to review the medical notes of patients listed for surgery on the dates that rifampicin vials were removed from the ADC; however, it is possible that some patients with documented use may have been missed.

## Conclusion

Around a quarter of Australian hospitals contributing data to national surveillance use rifampicin vials in the surgical setting, despite limited evidence of safety or efficacy. Given the potential for resistance and drug interactions, rifampicin use in the OT should be restricted to a named-patient basis, under the guidance of specialist infectious disease physicians or clinical microbiologists. Additionally, further research is required to determine the rate of systemic absorption of rifampicin when applied directly into a surgical wound. To assist AMS in the theater setting, governance processes should ensure that patient names are recorded when restricted antimicrobials are removed from ADCs. Documentation of any rifampicin use during a surgical procedure should be documented in the patient notes to minimize risks to patient safety with regard to interactions and side effects and to enable audits of patient outcomes.
